# Elevated c-Src and c-Yes expression in malignant skin cancers

**DOI:** 10.1186/1756-9966-29-116

**Published:** 2010-08-27

**Authors:** Jang Hyun Lee, Jae-Kyung Pyon, Dong Wook Kim, Sang Han Lee, Hae Seon Nam, Chul Han Kim, Sang Gue Kang, Yoon Jin Lee, Mi Youn Park, Dong Jun Jeong, Moon Kyun Cho

**Affiliations:** 1 Department of Plastic and Reconstructive surgery, Hanyang University Guri Hospital, Guri, Korea; 2 Department of Plastic and Reconstructive surgery, School of medicine, Sungkyunkwan University, Seoul, Korea; 3 Molecular Cancer Research Center, College of Medicine, Soonchunhyang University, Chunan, Korea; 4 Department of Dermatology, National Medical Center, Seoul, Korea; 5 Department of Dermatology, College of Medicine, Soonchunhyang University, Seoul, Korea

## Abstract

**Abstracts:**

**Background**

Src family kinases (SFKs) play an important role in cancer proliferation, survival, motility, invasiveness, metastasis, and angiogenesis. Among the SFKs, c-Src and c-Yes are particularly over-expressed or hyper-activated in many human epithelial cancers. However, only a few studies have attempted to define the expression and role of c-Src and c-Yes in cutaneous carcinomas.

**Objectives**

To investigate the expression of c-Src and c-Yes in cutaneous carcinomas to include malignant melanoma (MM), squamous cell carcinoma (SCC) and basal cell carcinoma (BCC).

**Methods**

We examined 6 normal skin tissues and 18 malignant skin tumor tissues using western blotting for the expression of c-Src and c-Yes. In another set, 16 specimens of MM, 16 SCCs and 16 BCCs were analyzed for the expression of c-Src and c-Yes using immunohistochemical staining.

**Results**

Western blotting showed that c-Src was expressed in all malignant skin tumors, but not in normal skin, while c-Yes was expressed in MM and SCC, but not in BCC and normal skin. Immunohistochemical staining results of c-Src and c-Yes in MM, SCC, and BCC mirrored those of the western blot analysis.

**Conclusions**

c-Src, rather than c-Yes, plays a key role in the proliferation and progression of malignant skin cancers.

## Introduction

Skin tumors have become one of the most common cancers in many countries, with rapid increasing incidence during the last half century. Nonmelanoma skin cancers including basal cell carcinoma (BCC) and squamous cell carcinoma (SCC) now make up more than one third of all cancers in the United States [1]. A large number of studies regarding the role of oncogenes and hormones, as well as environmental and predisposing factors, have been reported.

Oncogenes, especially Src family kinases (SFKs), which are activated in colon and breast cancers, are drawing attention for their involvement in malignant melanoma (MM) [2]. SFKs are non-receptor tyrosine kinases that participate in variable cellular signal transduction pathways, with the capacity to trigger cancer with its continuous activation. SFKs are composed of 9 members, c-Src, c-Yes, Fyn, Lyn, Lck, Hck, Blk, Rgr, and Yrk. SFKs play integral roles in cancer development to include proliferation, survival, motility, invasiveness, metastasis, and angiogenesis. Most SFKs are primarily expressed from a hematopoietic cell origin, but c-Src, c-Yes, and Fyn are expressed at high levels by platelets, neurons, and some epithelial tissues [3]. c-Src and c-Yes in particular are over-expressed or hyper-activated in many human epithelial cancers. The role and process of these two oncogenes in colon and breast cancer are well studied, but not in other human cancers. The role of SFKs in melanoma have been investigated with conflicting reports, but their overall role in nonmelanoma skin tumors has yet to be elucidated. Tyrosine kinases are known to be activated in many human MM, SCC, and BCC epithelial cancers. Therefore, we studied the expression of c-Src and c-Yes to uncover its involvement in malignant skin cancer development.

## Materials and methods

### Tissue samples

A total of 8 normal skin tissues and 24 malignant skin tumor tissues were obtained from patients who underwent surgery between July 2009 and September 2009 in the Departments of Plastic and Reconstructive Surgery at Hanyang University Guri Hospital and Soonchunhyang University Hospital in South Korea. Informed consent was obtained from the patients before surgery. The malignant skin tumor tissues, including 8 MM, 8 SCC, and 8 BCC, were obtained from patients who were treated with excisional surgery. All tumor tissues were examined using both conventional histopathological confirmation and immunohistochemical studies to confirm the diagnosis. Clinical and histopathological data are shown in Table 1. A portion of the specimens were frozen in liquid nitrogen immediately after resection and stored at -80°C degrees for subsequent western blot analysis. The human malignant melanoma cell line G361, obtained from the American Type Culture Collection (CRL 1424; Rockville, MD, USA), served as a positive control for c-Src and c-Yes expression.

**Table 1:** Clinicopathological features of 24 malignant skin tumors

Case	**No**.	Sex/Age	Site	Tumor type
1	M-1	F/53	Foot	MM(ALM)
2	M-2	F/51	Lower back	MM(NM)
3	M-3	M/70	Foot	MM(NM)
4	M-4	M/66	Foot	MM(NM)
5	M-5	M/54	Thigh	MM(ALM)
6	M-6	M/65	Thumb	MM(NM)
7	M-7	M/58	Foot	MM(ALM)
8	M-8	M/63	Foot	MM(SSM)
9	S-1	F/86	Temple	SCC
10	S-2	F/76	Cheek	SCC
11	S-3	M/51	Buttock	SCC
12	S-4	F/86	Face	SCC
13	S-5	F/87	Cheek	SCC
14	S-6	F/74	Scalp	SCC
15	S-7	F/82	Temple	SCC
16	S-8	F/77	Cheek	SCC
17	B-1	F/67	Cheek	BCC
18	B-2	M/75	Nose	BCC
19	B-3	M/52	Nose	BCC
20	B-4	M/64	Nose	BCC
21	B-5	F/68	Nose	BCC
22	B-6	F/71	Lower lid	BCC
23	B-7	F/65	Nose	BCC
24	B-8	M/56	Cheek	BCC

Abbreviations: MM, malignant melanoma; ALM, acral lentiginous melanoma; NM, nodular melanoma; SSM, superficial spreading melanoma; SCC, squamous cell carcinoma; BCC, basal cell carcinoma.

Levels of invasion of MM (M-1~M-7) were Clark's Level IV, M-8 was Level I.

### Western blot analysis

Tissue samples were homogenized in WCE buffer [25 mM HEPES (pH 7.7), 0.3 M NaCl, 1.5 mM MgCl_2_, 0.2 mM ethylenediamine tetraacetic acid (EDTA), 0.1% Triton X-100, 0.5 mM dithiothreitol (DTT), 20 mM-glycerolphosphate, 0.1 mM Na_3_VO_4_, 2 g per mL leupeptin, 2 g per mL aprotinin, 1 mM phenylmethylsulfonyl fluoride (PMSF), and a protease inhibitor cocktail tablet (Boehringer Mannheim)]. The tissue suspension was rotated at 4°C for 10 minutes. Supernatants were collected and then kept at -70°C and used for western blotting. Proteins from the tissue were separated by SDS-PAGE using NuPAGE 4-12% bis-Tris gels (Invitrogen, NP0335Box) and then transferred to Immobilon-P membranes. The membrane was blocked using 5% BSA in TBS-T (20 mM Tris, pH 7.6, 130 mM NaCl, and 0.1% Tween 20) solution. 6 MM, 6 SCC, 6 BCC and 6 normal skin tissues were then reacted with the primary antibody, Src (36D10) rabbit mAb (Cell Signaling technology^®^, #2109) and Yes antibody (Cell Signaling technology^®^, #2734) diluted to 1:1,000 concentration, at 4°C for 16 hours. This was then followed by washing buffer and TBST buffer washes (10 mM Tris-Cl, pH 8.0, 150 mM NaCl, 0.05% Tween 20), 4 times for 10 minutes, 10 minutes, 15 minutes and 15 minutes and then reacted with anti-rabbit IgG (Cell Signaling technology^®^, #7074) -horseradish peroxidase-linked species-specific whole antibody dilutes to 1:2,000 for 1 hour. After the reaction with the secondary antibody, it was washed 4 times for 10 minutes, 10 minutes, 15 minutes and 15 minutes. Proteins on the membrane were detected using an enhanced chemiluminescence solution kit (Amersham, UK). The membranes were stripped and reblotted with anti-actin antibody (catalog number sigma A5441). Western blotting analysis with mouse monoclonal antibody specific for phospho-Src (Calbiochem-Novabiochem, San Diego, CA) and mouse monoclonal antibody specific for phospho-Yes (WAKO, Osaka, Japan), were carried out on the 2 MM, 2 SCC, 2 BCC and 2 normal skin as described.

### Immunohistochemical staining

For immunohistochemical studies, the stored formalin-fixed, paraffin-embedded samples that included, 16 MM, 16 SCC, and 16 BCC were used. Parraffin sections (4 μm) were deparaffinized in xylene, rehydrated in 10 mM citrate buffer (pH 6.0), and then heated in a microwave oven for 15 minutes to restore antigens. To suppress endogenous peroxidase within the tissues, the samples were treated with 3% peroxide for 5 minutes, then with blocking solution for 30 minutes. Slides were incubated with primary Src (36D10) rabbit mAb and Yes antibody in a humid chamber for 60 minutes. Tissue staining was visualized with 3,3'-Diaminobenzidine (DAB) (ScyTek, USA) substrate chromogen solution.

### Assessment of western blot analysis

The amount of expression in western blotting was measured with TINA software (Version 2.10e). Measured amount of expression of malignant skin tumors was compared to that of normal skin.

### Statistical analysis

The data from the TINA score were analyzed using the nonparametric Mann-Whitney test. A p < 0.05 was considered statistically significant.

## Results

### Western blot analysis

**Figure 1:**
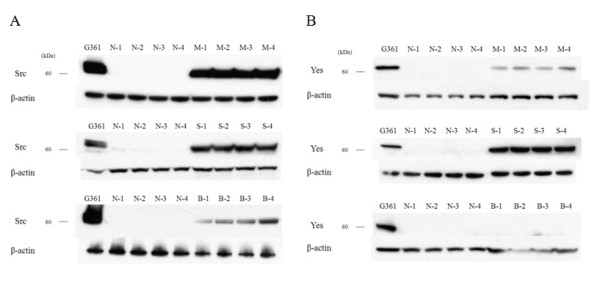
**Western blot analysis for c-Src and c- Yes in malignant skin tumor and normal skin**. (A) c-Src was expressed in malignant melanomas (MM) (M-1 - M-4), squamous cell carcinomas (SCC) (S-1 - S-4) and basal cell carcinomas (BCC) (B-1 - B-4), but not in normal skin (N-1 - N-4). (B) c-Yes was expressed in MM, SCC, but not in BCC and normal skin.

**Figure 2:**
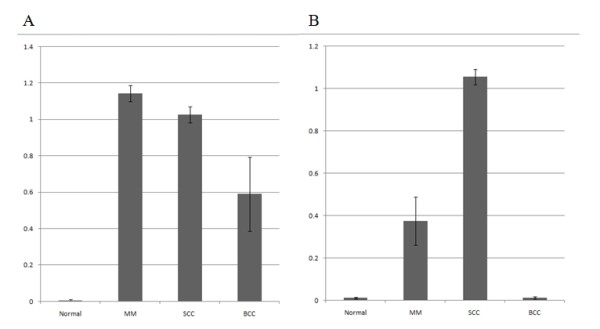
**The score of expression amount using western blotting**. (A) c-Src, (B) c-Yes.

**Figure 3:**
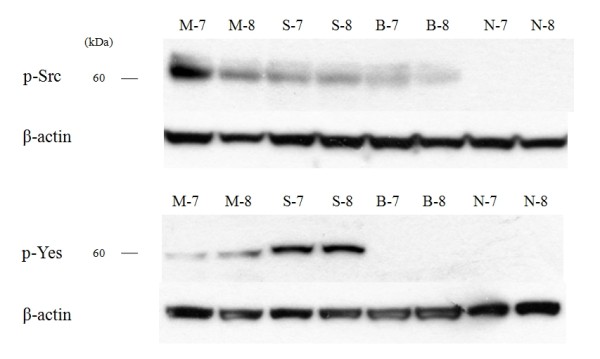
**Western blot analysis for phospho-Src and phospho-Yes in malignant melanoma (M-7, M-8), squamous cell carcinoma (S-7, S-8), basal cell carcinoma (B-7, B-8) and normal skin (N-7, N-8)**. The expression pattern of the phosphate form mirrored that of the total form.

Western blot analysis was performed to determine the expression of c-Src and c-Yes in 18 malignant skin tumors and 6 normal skin tissues. c-Src was expressed in all malignant skin tumors but not expressed in normal skin tissues, while c-Yes was expressed in MM and SCC and not in BCC and normal skin (Fig. 1) (data not shown for M-5, M-6, S-5, S-6, B-5, B-6, N-5, N-6). The expression amount score in western blotting was measured by TINA software (Version 2.10e), and the average TINA score of c-Src was 0.006 in normal skin, 1.143 in MM, 1.027 in SCC, and 0.590 in BCC. The average TINA score of c-Yes was 0.011 in normal skin, 0.374 in MM, 1.054 in SCC, and 0.012 in BCC. There were significant differences in the TINA scores of c-Src between malignant skin tumors and normal skin (p = 0.002). Of the tumor tissues, significant differences in c-Src score among the tumors (p = 0.002) were noted. With regard to c-Yes expression, there were significant differences in the TINA scores between two skin tumors (MM and SCC) and normal skin (p = 0.002), and there was no significant difference between BCC and normal skin (p = 0.818). The expression amount score based on western blotting is graphed in Fig. 2. To confirm the expression in phosphate form, western blot analysis with phospho-Src and phospho-Yes was also performed in 2 MM, 2 SCC, 2 BCC and 2 normal skin tissues. Phospho-Src was expressed in all malignant skin tumors and not expressed in normal skin tissues and phospho-Yes was expressed in MM and SCC but not in BCC and normal skin (Fig. 3).

### Immunohistochemical examination

**Figure 4:**
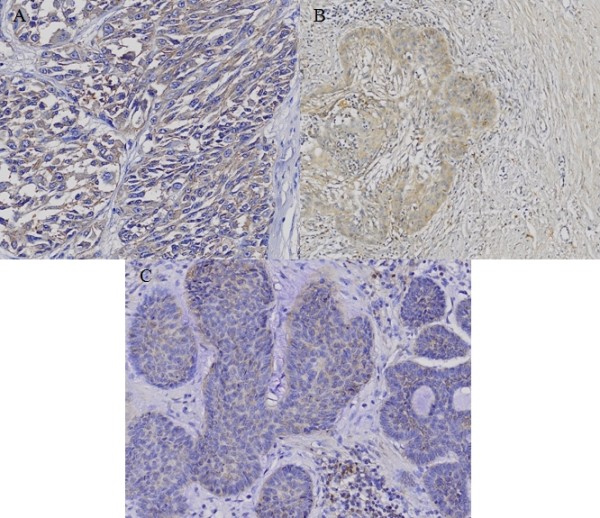
**Immunohistochemical staining of c-Src in (A) malignant melanoma (MM), (B) squamous cell carcinoma (SCC) and (C) basal cell carcinoma (BCC)**. c-Src protein is expressed in MM and SCC with moderate positivity, and BCC with mild positivity.

**Figure 5:**
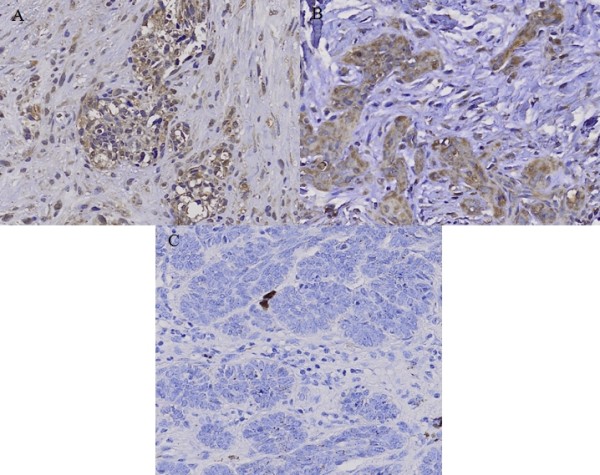
**Immunohistochemical staining of c-Yes in (A) malignant melanoma (MM), (B) squamous cell carcinoma (SCC) and (C) basal cell carcinoma (BCC)**. c-Yes was expressed in MM with moderate positivity and SCC with strong positivity, but was negative in BCC.

Immunohistochemical study showed that the staining pattern of c-Src and c-Yes in MM, SCC and BCC correlated with western blot analysis. c-Src protein was expressed in MM and SCC with moderate positivity, and BCC with mild positivity (Fig. 4). c-Yes was expressed in MM with moderate positivity and SCC with strong positivity, but not in BCC (Fig. 5).

## Discussion

The activation and functions of SFKs have been more investigated and better characterized in colon cancer and breast cancer compared to skin cancers. In colon cancer studies, c-Src protein level and kinase activity in the early-stages of colon cancer were found to be greater than in normal colonic mucosa [4,5]. The activity was highest in moderately to well-differentiated colonic lesions, while poorly differentiated carcinomas and normal colonic mucosa showed lower c-Src kinase activity [6]. Therefore, c-Src activity is directly related to the malignant potential of the cells, providing evidence that its activation contributes to the progression of colon cancer in the early and developing stages. SFKs also play an important role in the progression of breast cancers. SFK expression, as measured by immunoblotting with an antibody specifically recognizing Src, Fyn, and Yes, were elevated in 25 of 52 breast tumors. c-Src kinase and STAT3 activated hepatocyte growth factor expression in breast carcinoma cells [7,8]. Enhanced c-Src activity is also one potential mechanism leading to tamoxifen-resistant growth in breast cancer, and activation of c-Src and Fak has a close relationship with distant recurrence in hormone-treated, ER-positive breast cancer [9]. In recent studies, elevated c-Src activity was directly involved in the disruption of cell-cell adhesions in tamoxifen-resistant breast cancer cell lines, indicating that activated c-Src plays a role in the mislocalization of adhesion proteins [10]. Therefore, c-Src and c-Yes play important roles in colon cancer and breast cancer.

However, a very small number of studies have been conducted on SFK expression in skin cancer, and there is some controversy as to whether c-Src or c-Yes affects melanoma. By measuring tyrosine-specific kinase activity for c-Src expression in human melanoma tissues kinase activity in melanoma was found to be greater than that in normal skin regardless of the type of melanoma or the metastatic site [11]. In one study, Src kinase inhibitor dasatinib inhibited melanoma cell migration and invasion by inducing cell cycle arrest and apoptosis [12]. STAT3, which has been shown to play an important role in tumor cell proliferation and survival, and c-Src tyrosine kinase are activated in melanoma cell lines. Melanoma cells undergo apoptosis when either Src kinase activity or STAT3 signaling is inhibited [13]. This supports the fact that Src activated STAT3 signaling has a key role in the survival and growth of melanoma tumor cells. c-Src activation also affects epidermal growth factor of STAT in head and neck SCCs and promotes the invasion and progression of SCC [14-16].

On the contrary, it has been reported that c-Yes expression and kinase activity in human melanoma cell lines are greater than that in normal melanocyte cell lines, and that c-Src expression and activity are not different in human melanoma cell lines compared to normal melanocyte cell lines [17]. Similarly, it was demonstrated in another study that c-Yes tyrosine kinase was activated more in human brain-metastatic melanoma cell lines by stimulation of neurotropin and nerve growth factor, whereas c-Src was not affected [18]. These results show that c-Yes is more important than c-Src in melanoma progression and metastasis. Therefore, we studied the expression of both c-Src and c-Yes in overall human skin cancer tissues including MM, SCC, and BCC using western blotting and immunochemistry. Our study results show that c-Src was expressed in all skin cancer tissues, but not in normal skin tissues. c-Yes was expressed in MM and SCC, but not in normal skin tissues or BCC. c-Src was found to be significantly more expressed in MM, followed by SCC and BCC by both western blotting and immunohistochemistry, and c-Yes was intensely expressed in SCC. In general, MM is the most invasive of the skin tumors, followed by SCC and BCC. Given these facts, our results suggest that c-Src is expressed more in highly aggressive skin tumors, while c-Yes is expressed more in SCC compared to other skin cancers. We also confirmed that the expression pattern for phosphate Src and Yes forms in skin cancers were similar to the total forms. Therefore, we believe that c-Src, rather than c-Yes, plays a key role in the proliferation and progression of malignant skin cancers.

## Abbreviations

MM: Malignant melanoma; SCC: Squamous cell carcinoma; BCC: Basal cell carcinoma

## Competing interests

The authors declare that they have no competing interests.

## Authors' contributions

JHL, MKC designed the study and SHN, SHL, YJL carried out western blotting. DWK, MYP and DJJ carried out immunohistochemistry and JHL, MKC drafted the manuscript. J-KP, CHK, SGK participated in the manuscript drafting and performed the statistical analysis. All authors read and approved the final manuscript.
